# A Comprehensive Evaluation of Rejuvenator on Mechanical Properties, Durability, and Dynamic Characteristics of Artificially Aged Asphalt Mixture

**DOI:** 10.3390/ma11091554

**Published:** 2018-08-29

**Authors:** Pan Pan, Yi Kuang, Xiaodi Hu, Xiao Zhang

**Affiliations:** 1 Key Laboratory of Highway Construction and Maintenance Technology in Loess Region, Ministry of Transport, Taiyuan 030006, China; panp@wit.edu.cn; 2 Key Laboratory of Highway Construction and Maintenance Technology in Loess Region, Shanxi Transportation Research Institute, Taiyuan 030006, China; 3 School of Civil Engineering and Architecture, Wuhan Institute of Technology, Wuhan 430205, China; kuangyi1992@hotmail.com; 4 State Key Laboratory of Silicate Materials for Architectures, Wuhan University of Technology, Wuhan 430070, China

**Keywords:** artificially aged asphalt mixture, rejuvenator, durability, dynamic characteristics, overlay tester

## Abstract

In this study, the aged asphalt binder and mixture were laboratory prepared through short-term ageing testing and long-term ageing testing. Firstly, the effect of rejuvenator on physical properties of aged asphalt binders was investigated. In addition, a series of laboratory tests were performed to evaluate the influence of ageing and rejuvenator content on the mechanical properties, durability and dynamic characteristics of asphalt mixtures. Physical test results of asphalt binder testified that rejuvenator used can efficiently recover the aged asphalt binder. However, the effect of ageing and rejuvenator content exhibits different trends depending on the physical property tests conducted. Moreover, artificially aged asphalt mixture with rejuvenator has better ability to resist moisture damage and ravelling. In addition, the ITSR value is more suitable to evaluate the moisture susceptibility for asphalt recycling. Although rejuvenator improves the thermal cracking resistance and fatigue property of aged asphalt mixture, rejuvenated mixture shows greater modulus and inferior ability to resist reflective cracking than the unaged mixture. Moreover, rejuvenated mixture shows less dependence on frequency at high temperature regions and stronger dependence at low temperature regions compared to unaged and long-term aged mixtures.

## 1. Introduction

Asphalt mixture is widely used as a common road material for structural layers of pavement worldwide. During the service period, weather conditions and repeated load would deteriorate the mechanical performance and durability of the asphalt mixture, and then lead to some irreversible pavement distresses such as ravelling, rutting, cracking, etc. Removal of old pavement structure consequently produces a large amount of reclaimed asphalt pavement (RAP). For instance, more than 60,000,000 t of RAP material can be obtained annually in China [1]. To reduce the construction cost and generate environmental benefits, different countries have launched a series of experimental and practical researches on application of RAP material in new pavement construction [2,3].

Previous studies have confirmed that ageing of asphalt binder is the main cause of durability failure of asphalt pavement [4,5,6]. The ageing processes of asphalt pavement consists of short-term ageing during the mixing and construction phase, and long-term ageing during the service life in the field. There are many reasons that can lead to asphalt binder ageing including oxidation, volatilization, absorption by aggregate, thermal polymerization, etc. [7]. According to the molecular characteristics, asphalt binder can be divided into four fractions: saturates, aromatics, resins, and asphaltenes. From the chemical composition point of view, ageing effect increases resins and asphaltenes while reduces aromatics in RAP binder, compared to the virgin asphalt binder [8]. Due to the aged RAP binder, recycling asphalt pavement is prone to ravelling and low temperature cracking [9,10,11]. Based on the component control theory of asphalt recycling, soft binder is needed to reduce the asphaltene content and increase the aromatic content of the aged RAP binder, in order to minimize the negative impact of using RAP material in recycling asphalt pavement.

Rejuvenators, including plant oils, waster derived oils and engineered products, can provide an alternative method to restore the aged asphalt for hot asphalt recycling [12,13,14]. Many research studies have been conducted to evaluate the effectiveness of different types of rejuvenators on artificially aged asphalt binder or/and aged RAP binder. In this case, the aged binder is heated and blended with the rejuvenator. Chen et al. investigated the effect of waste edible vegetable oil on artificially aged asphalt binder. They concluded that waste edible vegetable oil can be used as a rejuvenator in recycling asphalt pavement [8]. Based on Superpave design specification, Zaumanis et al. developed a procedure to determine the optimum rejuvenator content for modifying RAP binder [15]. It is highlighted that considering ageing effect is helpful for better understanding the effect mechanism of rejuvenator on RAP binder [16]. Although the rejuvenator can recover the physical and rheological properties of aged asphalt binder to some extent, the rejuvenated asphalt binder is more prone to ageing than the virgin binder [17]. Moreover, both experimental and simulation results testified that the diffusion and distribution of rejuvenator play an important role in affecting the engineering performance of recycling asphalt mixture [18,19]. In a study conducted by Wang et al., the mortar transfer ratio (MTR) test was employed to study the diffusion characteristics of rejuvenator in aged asphalt [20]. 

By summarizing the present recycled asphalt techniques, Moghaddam et al. found that the most common way to use rejuvenator is mixing them with aged asphalt mixture at high temperature [2]. According to Chinese Specification, the rejuvenator is added and mixed with the reclaimed asphalt mixture, new aggregate and virgin asphalt to obtain the recycling asphalt mixture [21]. Therefore, it is worth noting that the actual production of hot asphalt recycling is quite different from that of the rejuvenated asphalt binder. Im et al. compared three different rejuvenators on engineering performance of asphalt mixtures containing recycles materials. They concluded that the effectiveness of rejuvenators prepared by different sources on recycled asphalt mixture depends on the engineering properties concerned [22]. 

For proper field usage of rejuvenator, it is of high priority to balance the cracking and rutting performance of recycled asphalt mixture. The rejuvenator should reduce fatigue and low temperature cracking potential whilst maintaining stability to rutting [23,24]. Therefore, it is necessary to obtain a comprehensive understanding of the engineering properties for rejuvenated asphalt mixture. Moreover, there is also a need for sufficient understanding of the dynamic characteristics of recycled hot mix asphalt. These problems of recycled hot mix asphalt with rejuvenator provide the motivation to undertake this research. 

In this study, a series of laboratory tests were conducted to investigate the influences of rejuvenator content on mechanical properties, durability and dynamic characteristics of artificially aged asphalt mixture. By studying the effect of using different amounts of a rejuvenator in recycled HMA and in aged binders subjected to different ageing times, this paper can not only clarify the effect of ageing process and rejuvenator content on the concerned properties of asphalt binder and HMA, but also demonstrate the suitability of available test or indicator to evaluate the performance of rejuvenated asphalt mixture. The finding of this study could help to gain a comprehensive understanding of rejuvenated asphalt mixture, and therefore contributes to proper usage of rejuvenator in recycled asphalt pavement.

## 2. Materials and Experimental

### 2.1. Materials

AH-90 paving asphalt with a penetration of 96 dmm (deci-millimetre, 25 °C), a softening point of 48.6 °C and a ductility of 156 cm was obtained from the Hubei Guochang Hi-tech Material Co., Ltd., Wuhan, China. The crushed diabase aggregate has a density of 2.953 g/cm^3^ and a particle size less than 16 mm. The limestone filler (LF) with a density of 2.699 g/cm^3^ was used as the mineral filler. The rejuvenator in this study was petroleum derived product, which was obtained from market. Table 1 illustrates the technical parameters of rejuvenator.

### 2.2. Sample Preparation

For asphalt binder, aged samples were prepared by the thin film oven test, TFOT (ASTM D1754) and the pressurized ageing vessel, PAV test (ASTM D6521). The detailed TFOT and PAV test procedures were described in a previous research work [25]. For the standard PAV test, asphalt binder sample is needed to be placed in the PAV for 20 h ± 10 min. To simulate different long-term ageing history, TFOT aged asphalt binders were treated by PAV test within 5 h, 10 h, 15 h and 20 h, respectively. Generally, 20 h of PAV ageing approximates 2 to 3 years of asphalt pavement in service. Another TFOT aged binder without PAV test was employed as a control sample. Then, four dosages of rejuvenator (2.0%, 4.0%, 6.0% and 8.0%, by weight of binder) were added into each TFOT and PAV aged asphalt binder. The rejuvenated binder was blended using the propeller mixer at a constant speed of 800 rpm for 20 min at 135 °C. All the aged and rejuvenated asphalt binders were used for physical properties tests in Section 2.3.

**Figure 1:**
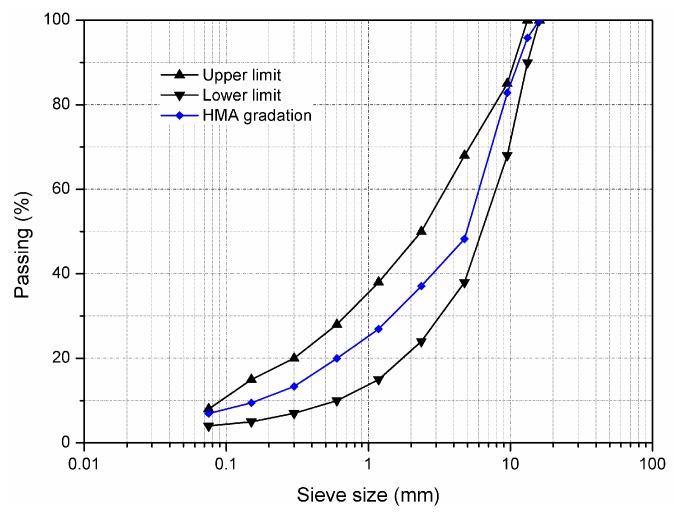
Chart of aggregate gradation.

Figure 1 shows the gradation curve of asphalt mixture with the nominal maximum size of 13 mm. The upper and lower limits of gradation followed to the Chinese Specification of JTG F40-2004 [26]. The content of mineral filler was 4% by weight of aggregate and the optimum asphalt content was 4.7% by weight of asphalt mixture. Aged asphalt mixtures were prepared by short-term ageing (STA) test and long-term ageing (LTA) test according to JTG E20 T0734 [27]. During the STA test, the incompact asphalt mixture was firstly spread to a height of 50 mm on a metal pan and placed in a force draft oven at 135 °C for 4 h. Then the compacted samples were prepared by STA aged mixture to perform LTA test, which were placed in the oven at 85 °C for 5 days. Finally, heated LTA aged sample was broken apart and then mixed with rejuvenator to prepare rejuvenated sample. All the unaged, LTA aged, and rejuvenated samples were used for mechanical properties, durability and dynamic modulus tests in Section 2.4.

### 2.3. Physical Properties Test of Asphalt Binder

The physical properties of the aged and rejuvenated asphalt binders, including softening point temperature, penetration (25 °C), ductility (15 °C), and rotation viscosity (135 °C) were studied in accordance with ASTM D36 [28], D5 [29], D113 [30] and D4402 [31], respectively.

### 2.4. Performance Test of Asphalt Mixture

#### 2.4.1. Moisture Susceptibility Test

Considering the soaking and frost effect, Marshall stability (MS) and indirect tensile strength (ITS) tests were conducted to evaluate the moisture susceptibility of aged and rejuvenated asphalt mixtures according to JTG E20 T0709 and T0729, respectively [27]. The detailed test protocols for Marshall stability and indirect tensile are shown in Table 2. Four repetitions were prepared for each group of the MS and the ITS tests. The loss of strength due to the impact of the exposure condition were defined as a ratio of the corresponding test result of conditioned samples to the control samples. The Marshall strength ratio (MSR) and indirect tensile strength ratio (ITSR) can be calculated as Equations (1) and (2):

(1) $$\mathrm{MSR}=\frac{\mathrm{MS}\;\mathrm{of}\;\mathrm{conditioned}\;\mathrm{samples}\;}{\mathrm{MS}\;\mathrm{of}\;\mathrm{control}\;\mathrm{samples}}\times 100\%$$

(2) $$\mathrm{ITSR}=\frac{\mathrm{ITS}\;\mathrm{of}\;\mathrm{conditioned}\;\mathrm{samples}\;}{\mathrm{ITS}\;\mathrm{of}\;\mathrm{control}\;\mathrm{samples}}\times 100\%$$

#### 2.4.2. Cantabro Abrasion Test

Since ravelling is a typical distress for rejuvenated mixture, the Cantabro abrasion test was performed to evaluate the resistance to particle loss of the aged and rejuvenated asphalt mixtures according to JTG E20 T0733 [27]. The compacted aged and rejuvenated samples were divided into control and conditioned groups. Before testing, the conditioned samples were kept in a water bath of 20 °C for 20 h. Then, four repetitions for each group were individually put in the Los Angeles abrasion device without steel balls. The abrasion test would be stopped when Los Angeles drum rotated for 300 revolutions at a speed of 30–33 revolutions per minute. After testing, the loose particles broken off from the surface of the testing sample were discarded. The mass loss ratio of sample before and after the test was adopted to evaluate the resistance to particle loss of mixture, which can be calculated as Equation (3):

(3) $$\mathrm{Mass}loss=\left(1-\frac{sample\;mass\;after\;test\;}{sample\;mass\;before\;test}\right)\times 100\%$$

#### 2.4.3. Wheel Tracking Test

The wheel tracking test was employed to evaluate the resistance to permanent deformation of the aged and rejuvenated asphalt mixtures under dry condition. The size of test sample is 300 mm in length, 300 mm in width and 50 mm in height. Before testing, the aged and rejuvenated samples were kept in the test device of 60 °C for 6 h. The repeated load was 0.7 MPa at 60 °C and the wheel speed was 42 passes per minute. The rutting depth of samples during the testing process was recorded. Dynamic stability can be calculated by the rutting depth of 45 min and 60 min as Equation (4) according to JTG E20 T0719 [27].

(4) $$\mathrm{DS}=\frac{15\times 42\;}{RD_{60}-RD_{45}}$$

where DS is dynamic stability, times/mm; RD_45_ and RD_60_ are the rutting depth at 45 and 60 min respectively, mm.

#### 2.4.4. Dynamic Uniaxial Compression Test

Dynamic uniaxial compression test was also carried out to assess the high temperature stability of the aged and rejuvenated asphalt mixtures according to BS EN 12697-25 [32]. Test samples with a diameter of 100 mm and a height of 110 mm were prepared by coring and cutting the SGC samples. Before testing, the aged and rejuvenated samples were kept in the test device of 60 °C for 4 h. A haversine load with a peak stress of 0.7 MPa was applied on the sample for 3600 s at 60 °C.

#### 2.4.5. Three-Point Bending Test

Three-point bending test was employed to evaluate the low temperature performance of the aged and rejuvenated asphalt mixtures according to JTG E20 T0715 [27]. The beam samples with 250 mm in length, 30 mm in width and 35 mm in height, were tested by a loading of 50 mm/min at −10 °C. Before testing, the samples were kept in the test device of −10 °C for 8 h. Four test repetitions were prepared for each mixture and the average value was used. The flexural strength, flexural strain, and flexural stiffness modulus were calculated by following equations:

(5) $$R_{B}=\frac{3LP_{B}\;}{2bh^{2}}$$

(6) $$\varepsilon_{B}=\frac{6hd\;}{L^{2}}$$

(7) $$S_{B}=\frac{R_{B}\;}{\varepsilon_{B}}$$

where P_B_ is peak load at failure, kN; R_B_ is flexural strength, MPa; ε_B_ is flexural strain; S_B_ is flexural stiffness modulus, MPa; L, b and h are length, width and height of sample, respectively, mm.

#### 2.4.6. Overlay Tester

The Overlay Tester (OT) was conducted to evaluate the reflective cracking resistance of the aged and rejuvenated asphalt mixtures according to TxDOT standard specification Tex-248F [33]. In this study, the OT samples were placed vertically and tested by UTM-100 at 25 °C. OT samples with 150 mm in length, 75 mm in width and 38mm in thickness were prepared by cutting the SGC samples. The triangular load with a constant maximum displacement of 0.625 mm was applied for 10 s, including a loading time of 5 s and a rest time of 5 s. 

#### 2.4.7. Four-Point Bending Fatigue Test

Four-point bending test was carried out to evaluate the fatigue performance of the aged and rejuvenated asphalt mixtures according to JTG E20 T0739 [27]. The beam samples, with 380 mm in length, 65 mm in width and 50 mm in height were tested at four strain levels, including 400 με, 500 με, 600 με and 700 με. For each strain level, four test repetitions were prepared for each mixture and the average value was used. The frequency of cyclic loading was 10 Hz and the test temperature was 25 °C. The loading cycles is defined as fatigue life when the stiffness modulus was decreased by 50% compared to the initial modulus. Previous studies found that fatigue life of asphalt mixture to strain follows the power function, which is shown as Equation (8) [34,35].

(8) $$N_{f}=A{\left(1/\varepsilon \;\right)}^{n}$$

where N_f_ is the fatigue life of asphalt mixture, ε is the strain level, A and n are the regression coefficients.

#### 2.4.8. Dynamic Modulus Test

The dynamic modulus test was performed to study the viscoelastic characteristics of the aged and rejuvenated asphalt mixtures according to AASHTO TP62-07 [36]. The samples were tested by a haversine axial compressive stress with six frequencies ranging from 0.1 to 25 Hz at five temperatures including −10 °C, 4.4 °C, 21.1 °C, 37.8 °C and 54.4 °C. In accordance with a previous study, generalized logistic sigmoidal function was adopted to obtain the master curve of the dynamic modulus, which is shown as below in Equation (9) [37].

(9) $$\log\left|E^{*}\;\right|=\delta +\frac{\alpha }{{\left(1+\lambda \times e^{\beta +\gamma logf_{r}}\right)}^{\frac{1}{\lambda }}}$$

where |E*| is the dynamic modulus, MPa; f_r_ is reduced frequency, Hz; δ is minimum modulus value; α is span of modulus values; β, γ and λ are shape parameters. The reduced frequency f_r_ is defined as Equation (10):

(10) $$f_{r}=f\times \alpha_{T}\;$$

where f is the loading frequency, Hz; α_T_ is the shift factor, which can be defined by Williams-Landel-Ferry (WLF) equation as follows [38].

(11) $$\log\alpha_{T}=\frac{-C_{1}\left(t-t_{0}\;\right)}{C_{2}+\left(t-t_{0}\right)}$$

where C_1_ and C_2_ are the model coefficients; t_0_ and t are the reference temperature and the test temperature, °C. 

## 3. Result and Discussion

### 3.1. Effect of Rejuvenator on Physical Properties of Aged Asphalt Binder

**Figure 2:**
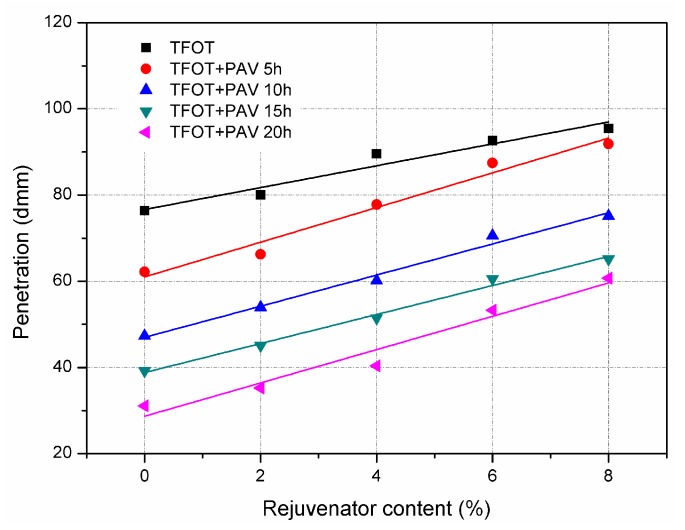
Penetration of TFOT and PAV aged asphalt binders with rejuvenator.

Figure 2 illustrates the effect of ageing process and rejuvenator content on penetration of asphalt binders. For the samples without rejuvenator, penetration of asphalt binders decreased with prolonging the ageing time. Since the PAV aged samples suffered longer ageing time compared to TFOT aged sample, penetration of four PAV aged asphalt binders were smaller than that of TFOT aged binder. For instance, penetration of TFOT aged binder was 76 dmm, while the corresponding results for 5 h and 20 h PAV aged binders were 62 dmm and 31 dmm, respectively. Penetration implies the stiffness of asphalt binder. The results indicate that ageing would make asphalt binders become much harder.

**Figure 3:**
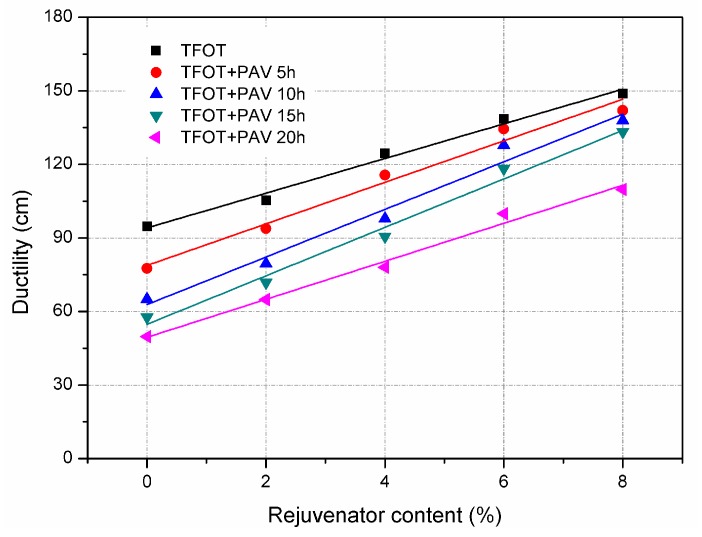
Ductility of TFOT and PAV aged asphalt binders with rejuvenator.

For each aged asphalt binder, penetration increased with increasing the addition of rejuvenator. When the rejuvenator was 8% by weight of asphalt binder, the penetration of 20 h PAV aged asphalt binder increased from 31 dmm to 61 dmm. The obtained result implies that rejuvenator can efficiently soften and recover the aged asphalt binder. It is also confirmed by the test result of ductility, which is shown in Figure 3. Incorporation of rejuvenator linearly increased the ductility of TFOT and PAV aged asphalt binders. Ductility is generally used to evaluate the resistance to low temperature cracking of asphalt binder. The greater ductility means the asphalt binder is more flexible. Therefore, rejuvenator helps the aged asphalt binder to be less susceptible to cracking in cold weather condition.

**Figure 4:**
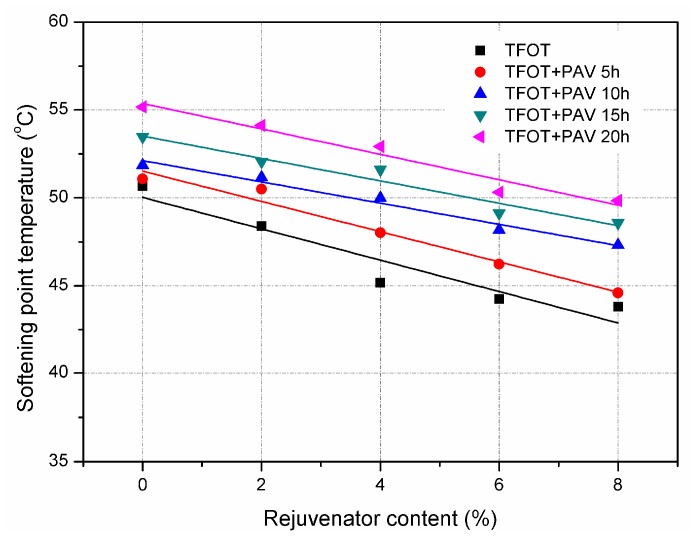
Softening point temperature of TFOT and PAV aged asphalt binders with rejuvenator.

**Figure 5:**
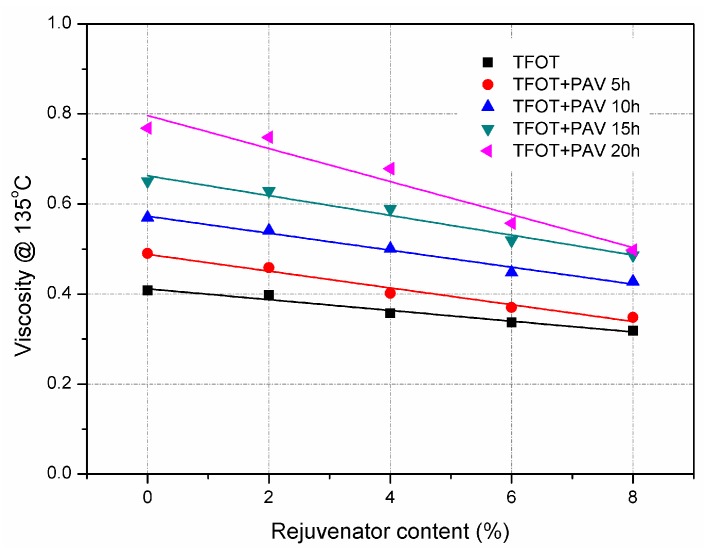
Viscosity of TFOT and PAV aged asphalt binders with rejuvenator.

Figure 4 and Figure 5 show the effect of rejuvenator on softening point temperature and viscosity of aged asphalt binders, respectively. Since asphalt binder would be harder after ageing, both the softening point temperature and viscosity increased due to the ageing effect. It can be assumed that aged asphalt mixture is less prone to permanent deformation at high temperature. For each aged asphalt binders, softening point temperature and viscosity shows a clear decline trend with increasing the rejuvenator content. It indicates that rejuvenator softened the aged asphalt binders, which is in accordance with the results of penetration and ductility. 

Results of physical properties confirmed that rejuvenator used in this study can restore the aged asphalt binder. Since rejuvenator modifies the flexibility of aged asphalt binder, the recycled asphalt mixture is expected to have a stronger ability to resist temperature cracking and fatigue cracking. Moreover, the addition of rejuvenator would decrease the softening point temperature and viscosity of aged asphalt binder. For instance, the softening point temperature of 5 h PAV asphalt binder with 8% rejuvenator was 44.6 °C, which was smaller than the Chinese specification of 48 ℃. It is worth noting that there is a risk of permanent deformation of recycled asphalt mixture with improper content of rejuvenator. Therefore, optimum rejuvenator content should be determined for obtaining recycled asphalt mixture with sufficient ability to resist both cracking and rutting.

Furthermore, it is noted that the changes in different physical properties of asphalt binder are quite different even after the same ageing process or adding the same content of rejuvenator into the aged asphalt binder. Compared to virgin asphalt binder, the penetration of 20 h PAV aged binder decreased 67.7% from 96 dmm to 31 dmm, while the corresponding softening point temperature increased 13.6% from 48.6 °C to 55.2 °C. By adding 8% rejuvenator, the penetration of 20 h PAV aged asphalt binder increased 96.8% from 31 dmm to 61 mm, and the softening point temperature accordingly decreased 9.8% from 55.2 °C to 49.8 °C. It can be concluded that the effect of ageing and rejuvenator content will exhibit different trends depending on the physical property test conducted. Future study is also suggested to examine the asphalt binder grade change due to the penetration and the softening point.

### 3.2. Effect of Rejuvenator on Engineering Properties and Durability of Aged Asphalt Mixture

#### 3.2.1. Moisture Susceptibility Test

**Figure 6:**
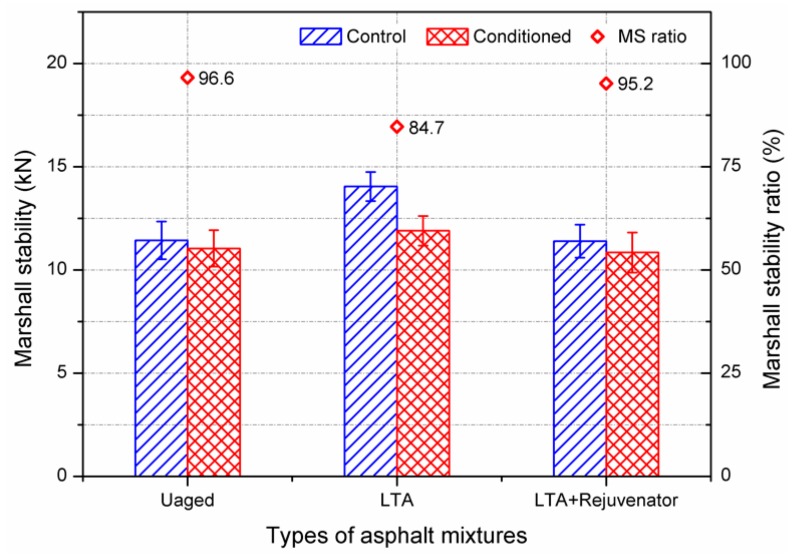
Marshall stability results of aged and rejuvenated asphalt mixtures.

Figure 6 shows the Marshall stability results of the unaged, long-term aged (LTA) and rejuvenated asphalt mixtures. Rejuvenated mixture was prepared by LTA mixture and rejuvenator. According to the aforementioned results in Section 3.1, the rejuvenator dosage was determined as 8% by weight of asphalt binder used in LTA mixture. The Marshall stability of control group for unaged and LTA samples were 11.43 kN and 14.04 kN, respectively. It indicates that long-term ageing process increased the stiffness of asphalt mixture. After soaking in a water bath of 60 °C for 48 h, the Marshall stability of unaged sample decreased to 11.04 kN, which was only a little lower the LTA sample of 11.89 kN. The Marshall stability ratio (MSR) greater than 80% is used as a critical indicator for hot mix asphalt. Although the MSR of LTA mixture was 84.7%, it is still quite lower than the unaged mixture of 96.6%. This implies LTA process makes asphalt mixture to be more susceptible to moisture. With incorporation of rejuvenator, rejuvenated asphalt mixture showed comparable Marshall stability and MSR value to the unaged mixture. It can be concluded that rejuvenator improves the resistance of aged asphalt mixture to moisture by the results obtained from Marshall stability test.

**Figure 7:**
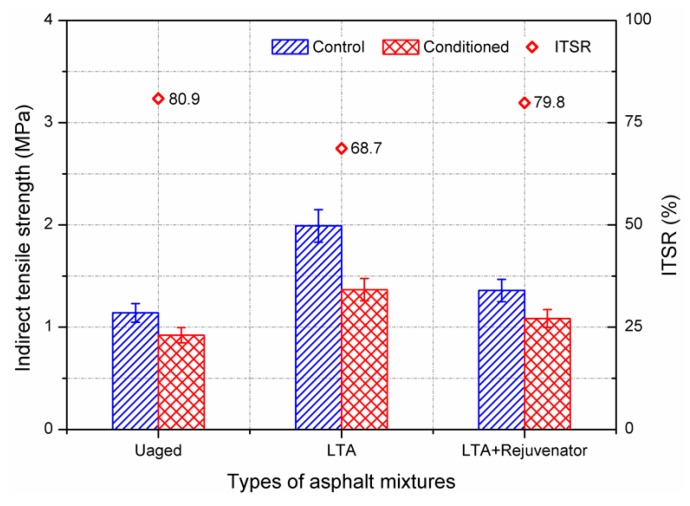
Indirect tensile strength test results of aged and rejuvenated asphalt mixtures.

In addition, indirect tensile strength test was also conducted to evaluate the moisture susceptibility of asphalt mixture, and the results are shown in Figure 7. Similar to MSR results, the indirect tensile strength ratio (ITSR) of rejuvenated sample was comparable to the unaged sample and greater than the LTA sample. ITSR results also confirmed the effectiveness of rejuvenator on aged asphalt mixture in term of moisture susceptibility. However, the indirect tensile strength of the control and conditioned rejuvenated samples were 1.36 MPa and 1.09 MPa, which were obviously greater than the corresponding results of unaged samples. Although the rejuvenator can recover the ability of aged asphalt mixture to resist freezing and thawing effect, it cannot enable the aged binder in LTA asphalt mixture to have a comparable stiffness with the unaged mixture at the test temperature of ITS. The reason might be that the softening effect of rejuvenator on aged mixture at low temperature for ITS test becomes less significant compared to that at high temperature condition for Marshall stability test.

Moreover, the ITSR value of LTA sample was 68.7%, which was lower than the performance requirement of 75% for hot mix asphalt according to the Chinese Specification of JTG F40-2004 [26]. For the indirect tensile strength test, freezing and thawing tests were performed on the asphalt mixture samples. Volume expansion and cracking would occur and lead to bonding failure between the asphalt binder and aggregate. The sample conditioning procedure is more efficient and serious than the Marshall stability test. Therefore, it is recommended to consider the ITSR value as a critical indicator for evaluating the moisture susceptibility, in order to reduce the risk of moisture damage for field usage of recycling hot mix asphalt.

#### 3.2.2. Cantabro Abrasion Test

**Figure 8:**
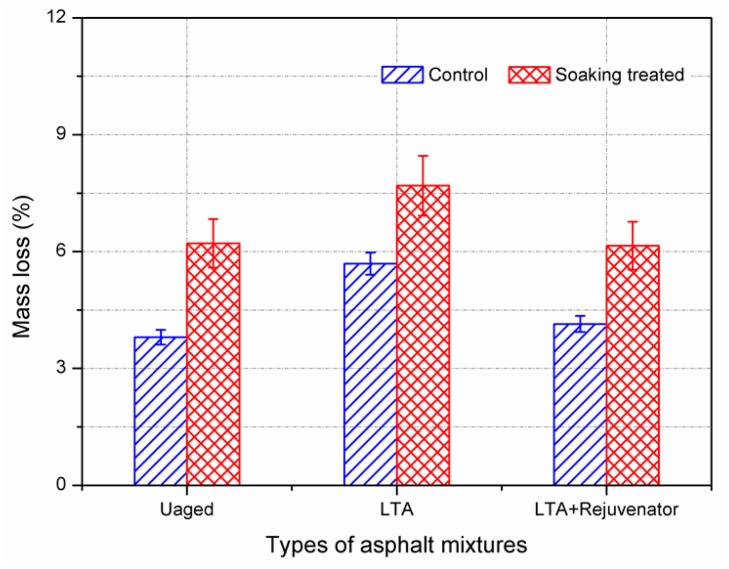
Cantabro abrasion test results of aged and rejuvenated asphalt mixtures.

In asphalt mixture, asphalt binder serves as a glue to bond aggregate. Ageing would degrade the bonding strength of asphalt binder and make asphalt mixture to be more prone to ravelling. Therefore, the Cantabro test, which is usually applied in open graded mixture, was performed to evaluate the effect of long-term ageing and rejuvenator on abrasion loss of asphalt mixture. Generally, lower abrasion loss indicates the tested sample has better resistance to ravelling. The results obtained from Cantabro abrasion test are shown in Figure 8. For the control samples, the abrasion losses for unaged and LTA were 3.82% and 5.69%. It implies that long-term ageing makes asphalt mixture more susceptible to ravelling. Moreover, the corresponding result of rejuvenated samples was 4.14%, which was lower than that of LTA mixture and comparable to the unaged mixture. It testified that the resistance to ravelling was enhanced by incorporation with rejuvenator into aged mixture. This might be attributed to the improvement of bonding strength between rejuvenated asphalt binder and aggregate, which is in accordance with the results in Section 3.2.1. 

For the same asphalt mixture, the abrasion loss of soaking treated samples was greater than that of the control group. There were similar increments in abrasion loss for the tested asphalt mixtures. After soaking treated, the increments of abrasion loss were 2.41%, 1.99% and 2.01% for unaged, LTA and rejuvenated samples, respectively. It is noted that the abrasion loss of rejuvenated samples after soaking treated was 6.15%, which was comparable to the corresponding result of control unaged mixture of 6.21%. In this circumstance, the result confirmed rejuvenator as an efficient additive to restore the resistance to particle loss of aged asphalt mixture. 

#### 3.2.3. High Temperature Performance

**Figure 9:**
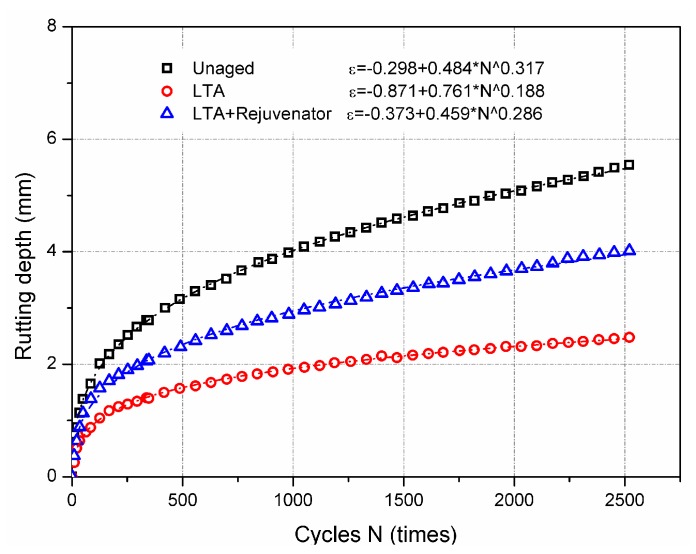
Wheel tracking test results of aged and rejuvenated asphalt mixtures.

Figure 9 illustrates the obtained results for the wheel tracking test. For the same testing time, rutting depth of LTA sample was the lowest, followed by the rejuvenated and the unaged samples. According to Equation (4), dynamic stability results of unaged, long-term aged (LTA) and rejuvenated asphalt mixtures were 1165 times/mm, 3088 times/mm, and 1898 times/mm, respectively. Greater dynamic stability means better high temperature performance of asphalt mixture. Due to the stiffening effect of ageing, LTA asphalt mixture after ageing has better ability to resist permanent deformation under repeated loading, compared to the unaged mixture. Since rejuvenator softens the aged asphalt binder, the rutting resistance of rejuvenated mixture was midway between the unaged and the LTA mixtures, which can be attributed to the combined effect of rejuvenator and ageing. 

Moreover, based on the test results the fitting equations of rutting depth and loading cycles were generated and are shown in Figure 9 as well. The equation parameters indicate that repeated loading is the least easily to induce the rutting for LTA mixtures, followed by the rejuvenated and unaged mixture. It can be concluded that softening effect of rejuvenator degrades the high temperature stability of asphalt mixture. It is noticeable that incorporation of rejuvenator into reclaimed asphalt mixture would increase the risk of permanent deformation for recycled asphalt pavement. 

**Figure 10:**
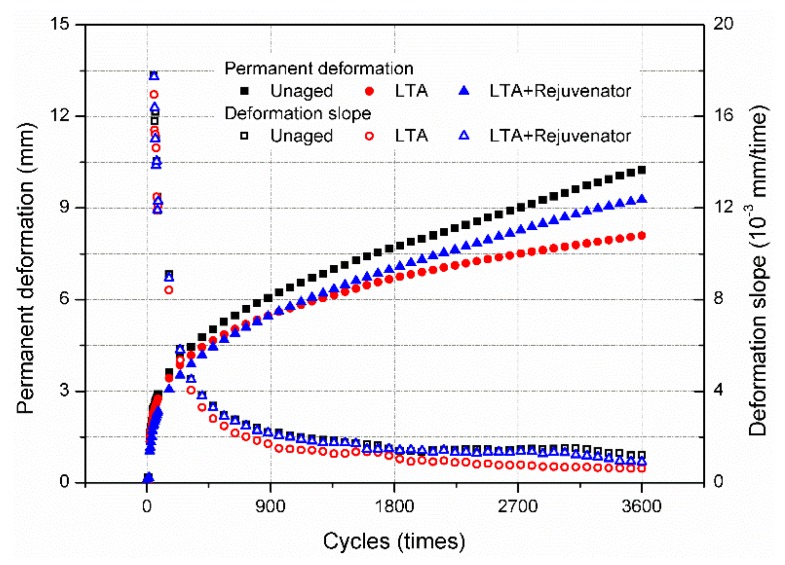
Dynamic uniaxial compression test results of aged and rejuvenated asphalt mixtures.

To simulate the actual traffic load, the dynamic uniaxial compression test was performed to assess the high temperature performance of asphalt mixtures and the results are shown in Figure 10. The samples were tested at 60 °C and 0.7 MPa stress. For the same loading cycles, the permanent deformation of rejuvenated mixture was greater than the LTA mixture and lower than the unaged mixture, which was in accordance with the results of wheel tracking test. By using differential method, the deformation slope for each mixture was calculated and is presented in Figure 10 as well. Lower slope means the asphalt mixture is less susceptible to permanent deformation. The deformation curves for the asphalt mixture can be divided into two stage: initial stage and stable stage. The deformation slope of rejuvenated sample was slightly lower than the unaged sample during the whole test period, and their deformation curves were almost parallel in the stable stage. Moreover, the deformation slope of LTA sample was obviously lower than the rejuvenated sample. It also implies that rejuvenator softens the aged asphalt binder and lowers the ability of LTA mixture to resist permanent deformation. 

#### 3.2.4. Cracking Resistance

**Figure 11:**
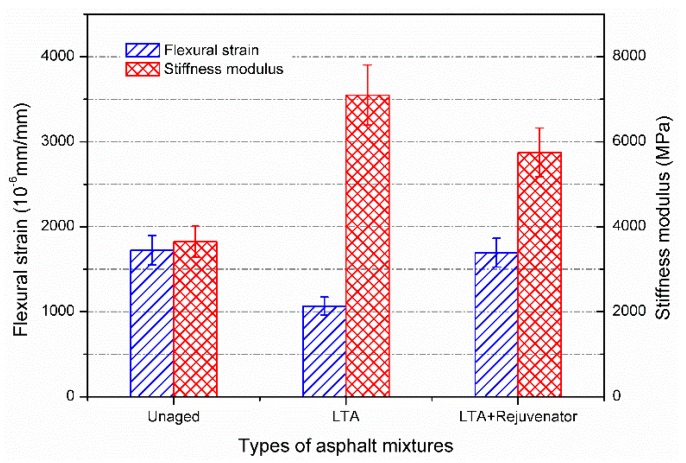
Three point bending test results of aged and rejuvenated asphalt mixtures.

Figure 11 illustrates the flexural stain and stiffness modulus of unaged, LTA and rejuvenated asphalt mixtures. Greater flexural stain means asphalt mixture has better ability to resist thermal cracking at low temperature. Compared to the unaged asphalt mixture, the flexural strain of LTA mixture decreased from 1724 με to 1066 με while the stiffness modulus correspondingly increased from 3467 MPa to 7095 MPa. It indicates that long-term ageing stiffened the asphalt mixture, resulting in inferior flexibility to low temperature cracking. Therefore, it is needed to improve the resistance to low temperature cracking of recycled asphalt pavement. By incorporating rejuvenator into LTA mixture, the flexural strain of rejuvenated asphalt mixture increased from 1066 με to 1695 με, which was comparative to the unaged mixture. The result indicates the rejuvenator could improve the ability to resist low temperature cracking for reclaimed asphalt pavement in term of flexural strain. However, it should be noticed that the stiffness modulus of rejuvenated asphalt mixture was still greater than that of unaged mixture. In the present study, the stiffness modulus of rejuvenated and unaged asphalt mixtures were 5748 MPa and 3647 MPa, respectively. From this point of view, the rejuvenator cannot fully restore the stiffness modulus of concerned LTA mixture to the same level of unaged mixture, although they have similar flexural strains.

**Figure 12:**
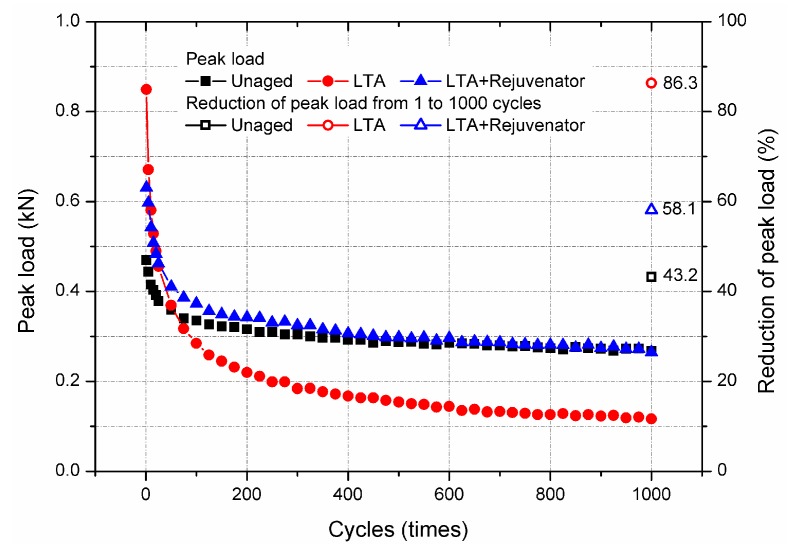
OT test results of aged and rejuvenated asphalt mixtures.

Overlay tester was also employed to investigate the cracking resistance at intermediate temperature of 25 °C for aged and rejuvenated asphalt mixtures and the obtained results are shown in Figure 12. The tensile force was applied to the sample and recorded for each load cycle. The peak load is strongly dependent on the modulus of asphalt mixture. Generally, OT samples with high modulus requires greater tensile force to keep the constant tensile displacement. As seen in Figure 11, the stiffness modulus of LTA mixture was the greatest, followed by the rejuvenated and unaged samples, respectively. Therefore, the peak load of LTA, rejuvenated and unaged sample presents the similar trend for the same loading cycle.

Moreover, the peak load decreased dramatically before almost 50 cycles and then changes little for all the asphalt mixtures concerned in this study. During the testing period, reduction of peak load was the result of a decrease in modulus of asphalt mixture, which contributes to the cracking formation and propagation in the tested sample. In term of this view, the decrement of peak load after 1000 cycles can be also adopted to evaluate the cracking resistance of asphalt mixtures. Greater reduction means the asphalt mixture is more prone to reflective cracking. After OT test, the reductions of peak load were 43.2%, 86.3% and 58.1% for unaged, LTA and rejuvenated samples, respectively. Rejuvenator can recover the reflective cracking resistance of LTA mixture, which was in accordance with the results in Figure 11. However, rejuvenated mixture showed slightly inferior resistance to reflective cracking compared with the unaged mixture. This might be attributed to the difference in stiffness modulus of rejuvenated and unaged mixtures. 

#### 3.2.5. Fatigue Property

**Figure 13:**
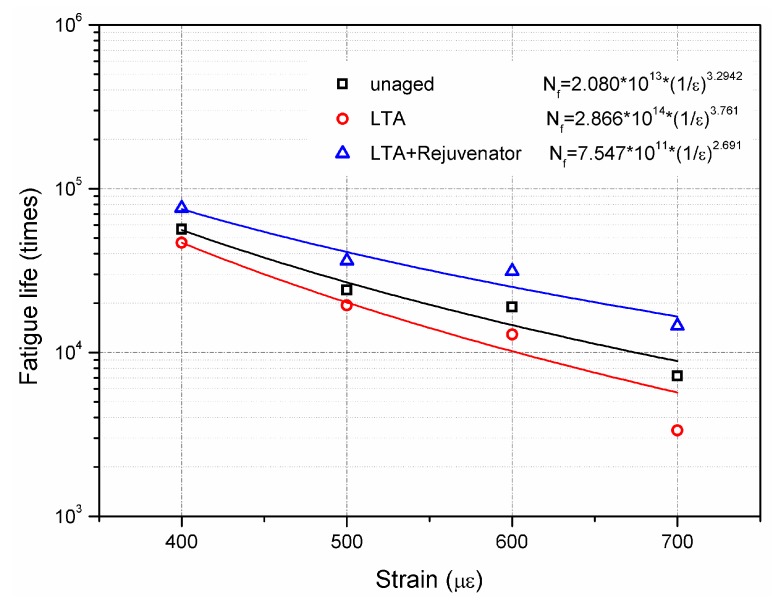
Four point bending fatigue test results of aged and rejuvenated asphalt mixtures.

Fatigue damage of asphalt pavement is caused by repeated stresses and strains due to traffic load. Figure 13 presents the fatigue life of unaged, LTA and rejuvenated asphalt mixtures under different strain levels including 400 με, 500 με, 600 με and 700 με, respectively. Compared to the fatigue life of unaged sample, there was significant improvement of fatigue life by incorporating rejuvenator into LTA asphalt mixture. Unlike the results in Section 3.2.4, rejuvenated mixture showed the most superior fatigue property within the whole testing strain levels. For OT test, a 2 mm gap between two bottom blocks was below the center of the sample. During the test period, stress concentration would induce the formation and propagation of reflective cracking. Compared to OT test result, the unexpectable results of fatigue test might be attributed to their different test mechanisms and load patterns applied. Nevertheless, it can be concluded that rejuvenator prolongs the durability of recycled asphalt pavement in terms of fatigue life. 

According to Equation (8), power function was used to analyze the test data and the fitting results are shown in Figure 13. Generally, greater n value represents stronger strain dependency of fatigue life. The n value of rejuvenated samples was 2.691, which was lower than the unaged and LTA samples. It indicates that the fatigue life of rejuvenated mixture changes less due to the strain variation compared to the unaged and LTA mixtures. For instance, the fatigue life of rejuvenated mixture decreased by 78.7% when the strain increased from 400 με to 700 με. The corresponding results for unaged and LTA mixture were 87.3% and 90.9%, respectively. It implies that rejuvenator leads the asphalt mixture to be less sensitive to strain level for the present fatigue test.

### 3.3. Dynamic Characteristics

**Figure 14:**
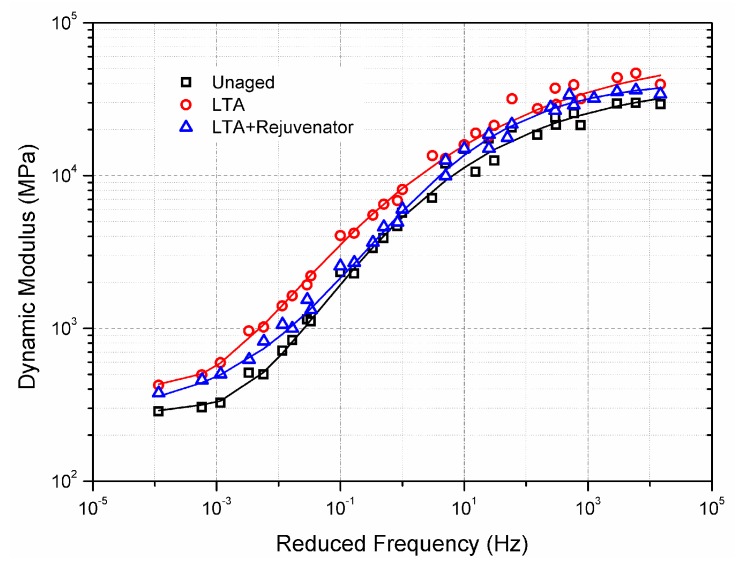
Master curves of dynamic modulus of aged and rejuvenated asphalt mixtures.

Master curves of dynamic modulus for unaged, LTA and rejuvenated asphalt mixtures are shown in Figure 14. The generalized logistic sigmoidal function can be fitted well with the test data of dynamic modulus. The dynamic modulus of rejuvenated mixture was greater than that of unaged mixture and lower than the LTA mixture within the whole reduced frequency region. This is attributed to the combined effect of long-term ageing and rejuvenator on asphalt mixture. Long-term ageing enhances the stiffness of asphalt mixture, while the rejuvenator softens the aged asphalt mixture. Although the addition of rejuvenator decreases the modulus of LTA mixture, rejuvenated mixture still showed greater dynamic modulus than the unaged mixture. This was also confirmed by the stiffness modulus results obtained in Section 3.2.4. 

It is interesting to note that rejuvenated mixture has similar dynamic modulus with LTA mixture at low frequency (high temperature) and at high frequency (low temperature) regions. In addition, the master curves for rejuvenated and unaged mixtures almost overlapped when the reduced frequency ranged from 0.1 Hz to 1 Hz. The result indicates that rejuvenated mixture shows less dependence on load frequency at relatively high temperature than both the unaged and LTA mixtures, and the corresponding dependence for low temperature condition is just the opposite. Further study is recommended to conducted on the effect mechanism of rejuvenator on frequency or temperature dependence of recycling asphalt mixture.

## 4. Conclusions

Laboratory ageing procedures were employed to prepare artificially aged asphalt binder and mixture. The effect of ageing and rejuvenator on asphalt binder and mixture was investigated by a series of laboratory tests in this study. Based on the research results, conclusions could be drawn as follows.

(1) Aged asphalt binder can be recovered to some extent by incorporating with rejuvenator. However, the effect of ageing and rejuvenator on asphalt binder differs from the physical properties concerned.
(2) Rejuvenated asphalt mixture has comparable resistance to moisture damage and ravelling. ITSR value is recommended as an indicator for evaluating the moisture susceptibility for recycling asphalt pavement.
(3) With similar flexural strain, rejuvenated asphalt mixture has greater modulus and inferior ability to resist reflective cracking than the unaged mixture.
(4) Rejuvenated asphalt mixture, which is less sensitive to strain level, shows better fatigue property than unaged mixture.
(5) Compared to unaged and LTA mixtures, rejuvenated mixture shows less dependence on frequency at high temperature regions and stronger dependence at low temperature regions.

The study compared the mechanical properties, durability, and dynamic characteristics of unaged, long-term aged, and rejuvenated asphalt mixtures by a series of available laboratory tests. Further study could be conducted on clarifying the rejuvenating mechanisms and effectiveness of different types of rejuvenators for reclaimed asphalt pavement. In addition, anti-ageing properties of rejuvenated asphalt mixture should be examined by both laboratory and field studies. Furthermore, critical indicators of rejuvenated asphalt mixture should be proposed to ensure the durability of recycled asphalt pavement. 

## Figures and Tables

**Table 1:** Technical parameters of rejuvenator.

Properties	Rejuvenator	Test Method
Form	Liquid	
Color	Brown	
pH	5.6	pH test paper
Viscosity (25 °C), SFS	15	ASTM D-244
Solute component (wt %)	60	ASTM D-244
Regeneration component (wt %)	8	ASTM D-2006-70
Asphaltene component (wt %)	0.75	

**Table 2:** Summary of moisture susceptibility test protocols used.

	Marshall Stability Test	Indirect Tensile Strength Test
Control	Soaking for 0.5 h at 60 °C	Soaking for 2 h at 25 °C
Conditioned	Soaking for 48 h at 60 °C	Freezing at −18 °C for 16 h + Thawing at 60 °C for 24 h + Soaking for 2 h at 25 °C
